# The Buffalo Street Car Nuisance Again

**Published:** 1890-02

**Authors:** 


BUFFALO MEDICAL AND SURGICAL JOURNAL
A MONTHLY REVIEW OF MEDICINE AND SURGERY.

*EDITORS:*
THOS. LOTHROP, M. D. - - - W. W. POTTER, M. D.
All communications, whether of a literary or business character, should be addressed to the editors: 284 FRANKLIN STREET, BUFFALO, N. Y.

### Editorial.

### THE BUFFALO STREET-CAR NUISANCE AGAIN.

WE HAVE on several occasions appealed to the Board of Health, of this city, to establish an inspection of the street cars, with a view to correct their unsanitary condition, and to put them upon the same basis as other public carriers. We need not go over the grounds of our complaint again. The nature of the offense that is daily perpetrated against common decency, as well as against the simplest rules of sanitation, is already well known to the street-car proprietors and to the Board of Health.

The citizens who patronize the street cars are, we fear, still in ignorance of the dangers they are exposed to, hence we return to the subject for the purpose of once more warning them thereof. We can do no more than this. We can only call attention to the perils of a ride in these cars in their present unhealthful condition. If the proprietors of the nuisance will not abate it of their own notion, and the Board of Health will not interfere to correct so grave an evil—one so threatening to health and comfort—then we can only advise those who would enjoy good health and long life to keep out of the street cars in winter.

We subjoin the following from the New York *Medical Record*, showing how the Philadelphia Board of Health exercises authority over this matter, and giving the opinion of an expert on the deleterious effects arising from the use of hay in the street cars:

HAY IN STREET CARS.—Medical Inspector Taylor, of Philadelphia, very properly declaims against the use of swamp hay in street cars as follows: "I have investigated the complaint about the use of swamp hay in the cars of one of the passenger railway companies, and find that, during wet weather, the hay becomes saturated with water, and loaded with the expectorations from scores of tobacco chewers, the sputa from tuberculous lungs, and street filth from passengers' shoes. The moist exhalations from this mass of filth are pernicious and prejudicial to health. If this hay is dried and used again the dust that is given off from the movements of the passengers finds lodgment in many delicate mucous membranes." Perfectly right, doctor; we agree with you in declaring the practice a public nuisance.

